# The Glutamate Dehydrogenase Pathway and Its Roles in Cell and Tissue Biology in Health and Disease

**DOI:** 10.3390/biology6010011

**Published:** 2017-02-08

**Authors:** Andreas Plaitakis, Ester Kalef-Ezra, Dimitra Kotzamani, Ioannis Zaganas, Cleanthe Spanaki

**Affiliations:** 1 Department of Neurology, Faculty of Medicine, School of Health Sciences, University of Crete, Heraklion, Crete 71003, Greece; esterkalefezra@gmail.com (E.K.-E.); dkotzamani@yahoo.gr (D.K.); johnzag@yahoo.com (I.Z.); kliospanaki@gmail.com (C.S.); 2 Icahn School of Medicine at Mount Sinai, One Gustave L. Levy Place, New York, NY 10029, USA

**Keywords:** GDH, hGDH1, hGDH2, structure, regulation, expression, human tissues, glioma, GDH deregulation and diseases

## Abstract

Glutamate dehydrogenase (GDH) is a hexameric enzyme that catalyzes the reversible conversion of glutamate to α-ketoglutarate and ammonia while reducing NAD(P)^+^ to NAD(P)H. It is found in all living organisms serving both catabolic and anabolic reactions. In mammalian tissues, oxidative deamination of glutamate via GDH generates α-ketoglutarate, which is metabolized by the Krebs cycle, leading to the synthesis of ATP. In addition, the GDH pathway is linked to diverse cellular processes, including ammonia metabolism, acid-base equilibrium, redox homeostasis (via formation of fumarate), lipid biosynthesis (via oxidative generation of citrate), and lactate production. While most mammals possess a single GDH1 protein (hGDH1 in the human) that is highly expressed in the liver, humans and other primates have acquired, via duplication, an hGDH2 isoenzyme with distinct functional properties and tissue expression profile. The novel hGDH2 underwent rapid evolutionary adaptation, acquiring unique properties that enable enhanced enzyme function under conditions inhibitory to its ancestor hGDH1. These are thought to provide a biological advantage to humans with hGDH2 evolution occurring concomitantly with human brain development. hGDH2 is co-expressed with hGDH1 in human brain, kidney, testis and steroidogenic organs, but not in the liver. In human cerebral cortex, hGDH1 and hGDH2 are expressed in astrocytes, the cells responsible for removing and metabolizing transmitter glutamate, and for supplying neurons with glutamine and lactate. In human testis, hGDH2 (but not hGDH1) is densely expressed in the Sertoli cells, known to provide the spermatids with lactate and other nutrients. In steroid producing cells, hGDH1/2 is thought to generate reducing equivalents (NADPH) in the mitochondria for the biosynthesis of steroidal hormones. Lastly, up-regulation of hGDH1/2 expression occurs in cancer, permitting neoplastic cells to utilize glutamine/glutamate for their growth. In addition, deregulation of hGDH1/2 is implicated in the pathogenesis of several human disorders.

## 1. The GDH Enzymes

**Figure 1:**
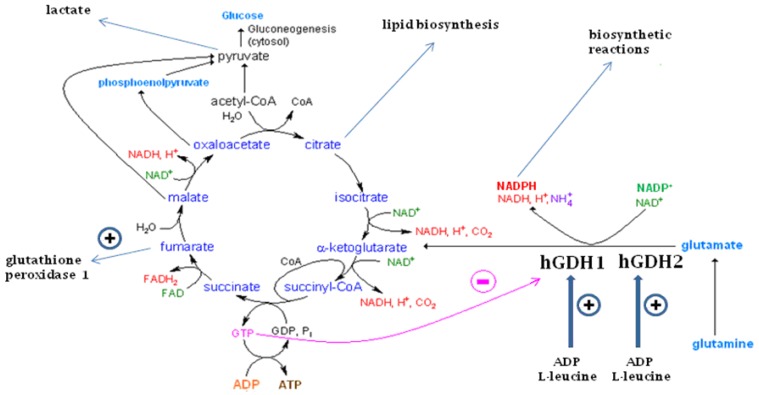
The glutamate dehydrogenase (GDH) pathway and the Krebs cycle function. As shown here, oxidative deamination of glutamate by hGDH1 and hGDH2 generates α-ketoglutarate, ammonia and NADH or NADPH. While α-ketoglutarate is metabolized by the Krebs cycle, NADPH can be used for biosynthetic reactions. GTP generated at the step of succinyl-CoA to succinate potently inhibits hGDH1 thus preventing glutamate from fueling this cycle under an adequate energy charge. On the other hand, hGDH2 has dissociated its function from GTP control and as such it permits glutamate flux under conditions inhibitory to hGDH1. Both enzymes are activated by ADP and L-leucine, which can act synergistically. Oxidative metabolism of α-ketoglutarate via the Krebs cycle generates citrate that can support the biosynthesis of lipids required for nerve tissue development and/or glioma cell growth. Also, it can generate lactate to be provided by astrocytes to neurons or by Sertoli cells to spermatids. Fumarate, generated by this pathway, is shown to stimulate glutathione peroxidase 1 activity, thus contributing to homeostasis against oxidative stress This Figure is modified from [7].

Glutamate dehydrogenase (GDH) is a hexameric enzyme that catalyzes the reversible conversion of glutamate to α-ketoglutarate and ammonia while reducing NAD(P)^+^ to NAD(P)H (Figure 1) [1]. In addition to contributing to Krebs cycle anaplerosis and energy production, GDH function is linked to redox homeostasis and cell signaling processes [2,3]. Lower life forms, such as bacteria or yeasts, often express distinct GDH isoenzymes that show strict specificity for NAD^+^ or NADP ^+^. The NAD^+^-dependent GDH serves mainly a metabolic role, whereas the NADP^+^-specific enzyme is involved in biosynthetic functions [4]. It has been argued that organisms that live in an environment rich in nutrient amino acids, use the NAD^+^-dependent GDH for their catabolic needs, including disposal of excess nitrogen [4]. On the other hand, organisms such as *Escherichia coli* capable of utilizing inorganic nitrogen (as nitrates or ammonia) use the NADP^+^-specific GDH for their synthetic needs [4]. In the yeast *Saccharomyces cerevisiae* three distinct GDH isoenzymes have been identified; yGDH1 (NADP^+^-specific), yGDH2 (NAD^+^-specific) and yGDH3 (NADP^+^-specific). While yGDH2 functions in the oxidative deamination of glutamate to α-ketoglutarate and ammonia, the NADP^+^-specific isoenzymes (yGDH1, yGDH3) are involved primarily in glutamate biosynthesis [5,6]. In lower organisms GDH is not modulated, with regulation being achieved at the transcription level.

GDH in plants exists in distinct isoenzymes that are either NAD^+^-specific or NADP^+^-specific [8,9,10,11]. The NAD^+^-dependent enzymes localize mainly, if not exclusively, to the mitochondria of the phloem companion cells [12,13,14]. In *Arabidopsis thaliana*, NAD^+^-specific GDH is encoded by three distinct genes (aGDH1, aGDH2 and aGDH3). These genes encode subunits for heterohexameric or homohexameric GDHs that are distributed either in the root and/or in the leaves [8]. In *Oryza sativa* (rice) NAD^+^-specific oGDH is also encoded by three distinct genes that show different expression pattern [15]. On the other hand, NADP^+^-specific GDH proteins have been identified in a range of higher plants. These localize to chloroplasts. In *A. thaliana* and of *O. sativa* the NADP^+^-specific GDH is 50% longer than the NAD^+^-specific enzymes. However, their biological function is not fully understood [8].

**Figure 2:**
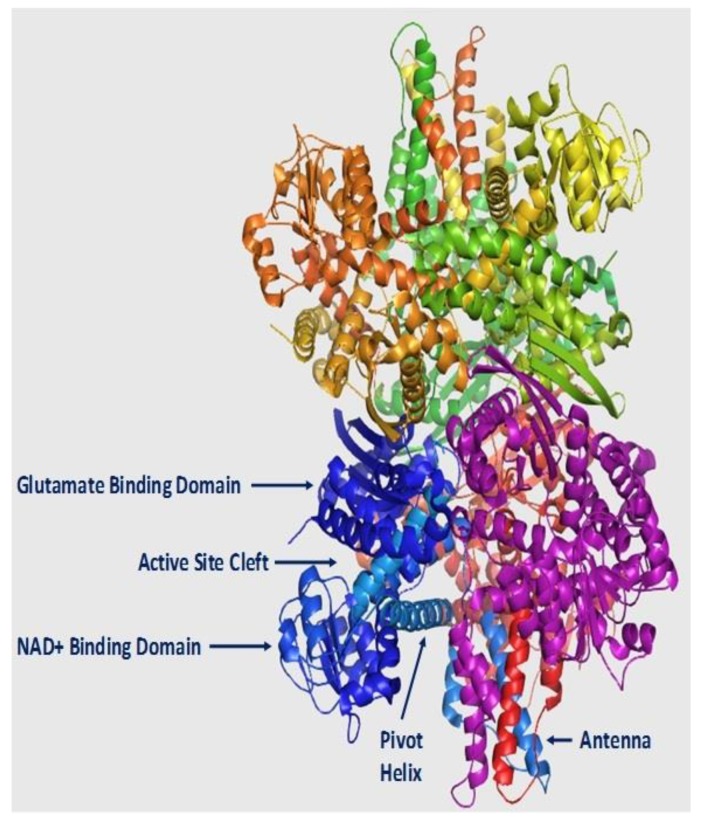
Structural model of hGDH1. Shown is a cartoon diagram of the apo form of the hGDH1 hexamer (PDB code: 1L1F), where each of the six subunits is colored differently. The main parts of the GDH subunit (active site, NAD^+^ binding domain, glutamate binding domain, antenna and pivot helix) are indicated only for the blue subunit. The figure was constructed using the PyMOL Molecular Graphics System, Version 1.8 (Schrodinger LLC., Cambridge, MA, USA).

During species evolution, GDH continued advancing to become in mammals a highly regulated enzyme with dual co-enzyme specificity [1,4]. The ability of mammalian GDH1 (hGDH1 in the human) to utilize both NAD^+^ and NADP^+^ enables the enzyme to play a role both in catabolic and in synthetic cellular processes. In addition, a sophisticated allosteric regulation system was acquired by GDH1. This provides a versatility needed for dynamic adaptation to diverse and changing cellular environments [16,17]. There is evidence that allosteric regulation emerged along with the appearance and maturation of the “antenna”, (Figure 2) a protruding structure that does not exist in the GDH of lower organisms [18,19,20] Mammalian GDH is composed of two trimers stuck one on top of the other. In the functional trimer, the antennas of adjacent subunits intertwine, with this subunit interaction mediating allostery [21]. Of the various allosteric effectors for mammalian GDH1, GTP and ADP are known to serve as the main endogenous negative and positive modulators, respectively. This allosteric regulation is thought to provide an energy-sensing mechanism by which GDH1 activity is controlled by the cellular needs for ATP [1,17]. Specifically, potent inhibition of the enzyme by GTP generated by the Krebs cycle is thought to link glutamate flux through the GDH1 pathway with the function of this cycle [17]. However, a novel GDH2 isoenzyme (hGDH2 in the human) that first appeared in primates has departed from this principle by dissociating its catalytic function from GTP control [22]. This enzyme multiplicity provides a new functional dimension to the GDH pathway that may be biologically advantageous (see below).

As noted above, while most mammalian species possess a single GDH1 enzyme that is widely expressed (housekeeping), humans and other primates have acquired via reduplication a second GDH-specific isoenzyme (hGDH2) with a distinct regulation and tissue expression profile. Whereas hGDH1 is encoded by the intron-containing *GLUD1* gene located on the 10^th^ human chromosome, hGDH2 is encoded by the intronless *GLUD2* gene that arose through retropositioning of *GLUD1* to the X chromosome [23,24]. The new hGDH2 isoenzyme acquired 15 evolutionary amino acid substitutions that provide unique properties [17,22]. Phylogenetic evidence suggests that *GLUD2* first appeared in the hominoid ancestor (<23 million years ago) and underwent rapid adaptation driven by positive selection [25]. An important functional adaptation, noted above, is resistance of hGDH2 to GTP inhibition via the evolutionary replacement of Gly456 by Ala [26] acquired shortly after the birth of the *GLUD2* gene [25]. These functional properties may provide a biological advantage to the human by permitting hGDH2 to operate independently of the Krebs cycle function. However, to prevent unregulated enzyme catalysis from perturbing cell metabolism, hGDH2 developed an alternate molecular mechanism, according to which the new enzyme down-regulates its basal activity (to about 4%–6% of its capacity) while remaining remarkably responsive to full range activation by rising ADP and/or L-leucine levels [22,27]. As described below, this adaptation is mainly mediated by the Arg443Ser change [28], subject to modification by other evolutionary amino acid substitutions [27]. There is increasing evidence that the GDH pathway serves diverse functional roles and that its complex tissue expression pattern supports these roles as discussed below [16].

## 2. Structure

Mammalian GDH has been thoroughly studied, both at the functional and the primary structure level, with the amino acid sequence of the protein having been determined prior to the advent of the molecular cloning techniques. More than half a century ago, GDH1 preparations, purified from mammalian tissues, became readily available through commercial sources, thus propelling research efforts on this protein [1,4]. Extensive functional analyses generated a wealth of data on the mechanisms used by this dehydrogenase for its catalytic function and regulation [4]. More recently, the use of molecular biologic approaches resulted in rapid progress in characterizing the genes encoding the GDH of different organisms and their products, including the two human GDH isoenzymes [23,29]. In addition, site directed mutagenesis and crystallographic studies have advanced our understanding of the molecular mechanisms utilized by GDHs of distinct genetic origin for their catalytic function and regulation [18,19,20,21,26,27,28].

At the structural level, GDH is a hexamer comprised of six identical subunits. Each of these polypeptides consists about 500 amino acids (molecular mass: ~56 kDa) [4,30]. The enzyme is composed of two trimers, with each trimer encompassing three main functional domains: the NAD^+^ binding, the glutamate binding and the regulatory domain (Figure 2). The regulatory domain includes the pivot helix and the “antenna”. The latter is a 48 amino acid segment that consists of an ascending α-helix, a random coil and a small C-terminal α-helix that shows striking conformational changes on opening and closure of the active site of the enzyme [18,19]. In the functional trimer, the antennas of the adjacent subunits are intertwined with this inter-subunit interaction thought to mediate allostery, but the precise mechanisms involved are not fully understood [21].

In recent decades, the solution of X-ray structures at high resolution from different GDHs has provided insight into the molecular mechanisms used by these enzymes to accomplish their catalytic function. Original observations on clostridial GDH revealed a simple “claw-like” appearance with a wide cleft separating the NAD^+^ and the glutamate domain of the enzyme [31,32]. In the presence of glutamate, the cleft largely closes due to side-chain movements that bring the two domains together. More recent studies on mammalian GDH1, an advanced structure that also encompasses the “antenna” as noted above, showed that the entire hexamer undergoes substantial conformational changes during each catalytic cycle [18,19,30]. It has been observed that as the catalytic cleft opens the NAD^+^ domain moves away from the glutamate binding domain, twisting around the antenna in a clockwise direction along with concomitant clockwise rotation of the ascending α-helix of the antenna. In addition, the small α-helix of the antenna (at the end of its descending random coil) undergoes striking conformational changes as the catalytic mouth opens [19,30]. The importance of this small helix is underscored by observations showing that mutation of amino acids located in this helix in hGDH1 attenuate GTP inhibition leading to hyperinsulinemia/hyperammonemia (HI/HA) syndrome [33].

## 3. The GDH Catalysis

Although GDH has been studied extensively over the past decades, its role in cell biology has not yet been fully elucidated. Extensive kinetic analyses have elucidated many aspects of GDH catalysis, although unsolved puzzles still remain [4]. It is known that the thermodynamic equilibrium of mammalian GDH favors glutamate synthesis, but it is presently unclear whether the enzyme operates in vivo towards the reductive amination or the oxidation-deamination direction. Because the GDH-catalyzed reaction is reversible, its direction is expected to depend on the concentration of the substrates and the affinity of the enzyme (Km value) for these substrates. In addition to substrate concentrations, the GDH catalysis is affected by the pH, the ionic strength and the composition of the buffer [4,27,34]. Thus, studies on GDH, purified from mammalian tissues or on recombinant hGDH1, have shown that the enzyme operates optimally at relatively basic pH (7.75 to 8.00) [27] and that ADP activation is markedly diminished at lower pH values [34]. On the other hand, the optimal pH for hGDH2 is 7.50, with the enzyme being able to operate efficiently at even lower pH values (7.25 to 7.0) [27]. When assayed in triethanolamine (TRA) buffer pH 8.0 at 1.0 mM ADP, hGDH1 and hGDH2 show similar catalytic properties (Vmax and Kms for α-ketoglutarate, ammonia and glutamate) [27]. Because of the relatively high Km for ammonia, GDH is expected to operate towards the oxidative deamination direction, particularly in tissues with normally low ammonia concentrations. There is evidence that the Km for ammonia is affected by cellular pH. Thus, using recombinant hGDH1, Zaganas et al. [35] recently showed that reducing the pH of the buffer from 8.00 to 7.5 or to 7.0 increases the Km for ammonia from 12.8 mM to 35.0 mM and 57.5 mM respectively. For recombinant hGDH2, the corresponding Km for ammonia changes from 14.7 mM to 33.0 mM and 62.2 mM [35]. These results suggest that intracellular acidification, as this may occur in astrocytes following glutamate uptake [36] or in the epithelial cells of the proximal convoluted tubules of the kidney during systemic acidosis, essentially precludes the reductive amination of α-ketoglutarate. Functional studies have indeed shown that under these conditions the GDH1 pathway is predominantly deaminating [37]. Of the divalent cations, present in mitochondrial matrix, Mg++ is shown to inhibit hGDH1/2 (at 1.0–2.0 mM) [38], whereas Ca++ has little effect on enzyme activity [39]. On the other hand, manganese (Mn++) proved to be more potent than Mg++ in inhibiting hGDH1 at 0.1 mM ADP [39]. However, in contrast to Mg++ that is normally present at 1.0 mM in mitochondrial matrix [40], Mn++ is only found in trace amounts in mammalian tissues. It is presently unclear, however, whether under conditions of Mn intoxication, the metal can attain levels in the mitochondrial matrix that are inhibitory to hGDH1/2 [39].

As noted above, a major evolutionary adaptation of hGDH2 is the ability of the enzyme to down-regulate its activity in the absence of allosteric effectors. This provided us with a natural model for studying the molecular mechanisms involved in basal enzyme catalysis. Thus, site-directed mutagenesis in hGDH1 at sites that differ in hGDH2 revealed that a single evolutionary amino acid substitution (Arg443Ser), located in the short helix of the descending chain of the antenna, diminishes basal catalytic activity and abrogates L-leucine activation [28]. Also, the Arg443Ser change renders the mutant enzyme heat-labile and increases its sensitivity to steroid hormones and neuroleptic agents. In addition, it shifts its optimal pH from 8.0 to 7.0 and its migration pattern in SDS-PAGE [28]. While these findings may explain, in part, some of the properties acquired by wild type hGDH2, substitution of Ser for Arg443Ser in hGDH1 is too disruptive, rendering the mutant enzyme essentially non-functional. Indeed, molecular modeling suggested that Arg443Ser interrupts hydrogen bonds between the 443 residue (in the small helix of the descending part of the antenna) of one monomer and the 409 residue (in the ascending helix of the antenna) of the opposing monomer. This may disrupt the antenna function during catalysis [41]. Additional mutagenesis studies support this hypothesis by showing that single amino acid replacement of the interacting Ser409 residue in hGDH1 has functional consequences similar to those of the Arg443Ser change. Moreover, a “swap” double mutation (S409R/R443S) (consisting in two inactivating amino acid replacements) restores basal activity and activation [41]. In light of these data, the Arg443Ser change cannot explain the catalytic properties of wild-type hGDH2. Instead, other amino acid substitutions acquired during hGDH2 evolution, should act in concert with Arg443Ser to provide the unique functional characteristics of hGDH2.

Additional functional analyses of human GDHs have shown that the basal activity of hGDH2 depends on the concentration of the recombinant enzyme present during assay [27]. Thus, increasing the concentration of the recombinant wild-type hGDH2 protein in the reaction cuvette is shown to augment its specific activity (R = 0.9862; *p* < 0.001). In addition to hGDH2, significant positive correlations between enzyme concentration and specific activity have been obtained for the single hGDH1 mutants Arg443Ser (R = 0.9890; *p* < 0001), Ser409Arg (R = 0.8728; *p* = 0.053) and Ser409Asp (R = 0.9586; *p* < 0.001), all of which display low basal activity. This is also true for the double Arg443Ser/Gly456Ala hGDH1 mutant (R = 0.9890; *p* < 0.0001) [27]. Dependence of the wild type hGDH2 and the above hGDH1 mutants on enzyme protein concentration is thought to relate to the disruption of the Arg443-Ser409 hydrogen bonds in the antenna [41]. These findings suggest that increased protein concentrations stabilize the enzyme. This property may be of functional relevance for the wild-type hGDH2, expected to attain high levels in the mitochondrial matrix of cells expressing this isoenzyme. In contrast to these observations on hGDH2 and hGDH1 mutants, varying the protein concentration of the wild-type hGDH1 has little effect on its specific activity. In fact, statistical analyses suggested a weak reverse correlation (R = 0.6009; *p* < 0.01) [41].

## 4. GDH Regulation

As described above, mammalian GDH is allosterically regulated, with chemically diverse compounds shown to influence the enzyme’s velocity. These include purine nucleotides (ADP, ATP and GTP) [34,42], NADH [34], L-leucine [43], palmityl CoA [44], spermidine [45,46], steroid hormones [47,48], neuroleptic drugs [49,50,51] and the green tea polyphenol epigallocatechin gallate (EGCG) [46,52]. Recently, the purpurin analogue R162 (2-allyl-1-hydroxy-9,10-anthraquinone) was identified as a potent inhibitor of GDH1 [3]. As noted above, GTP and ADP serve as the main endogenous negative and positive modulators for hGDH1, respectively, with this allosteric interaction thought to provide an energy-sensing mechanism by which enzyme activity is controlled by the cellular needs in ATP [1,17]. It appears that in some tissues, such as the pancreatic β-cells, hGDH1 is under tonic inhibition by GTP (generated by the Krebs cycle), with ADP and L-leucine counteracting GTP inhibition in a concentration-dependent manner.

While GTP potently inhibits hGDH1, it has little effect on hGDH2 [22]. On the other hand, ADP induces a proportionally greater activation of hGDH2 than hGDH1. Thus, ADP (at 1.0 mM) activates hGDH1 by 235% and hGDH2 by 1600% [27]. However, ADP shows a somewhat greater affinity for hGDH1 (SC_50_ = 25.0 μM) than for hGDH2 (SC_50_ = 54 μM). L-Leucine (0.5–5 mM) also induces a proportionally greater activation of hGDH2 than hGDH1, but with a similar affinity (SC_50_ = 1.0 mM for hGDH1 and 1.1 mM for hGDH2) [27]. These differences between ADP and L-leucine activation are thought to reflect the fact that hGDH2 evolution affected predominantly residues in the regulatory domain (where ADP binds) while sparing residues located in the catalytic site (where L-leucine is thought to bind). This concept is consistent with observations showing that, although hGDH1 and hGDH2 differ markedly in the regulation, they show similar catalytic properties.

Regarding the mode of action of the allosteric effectors GTP and ADP, these appear to act by affecting the NAD^+^ domain dynamics. Thus, GTP is shown to bind in the closed conformation at the base of the antenna (wedged between the NAD^+^ domain and the pivot helix), with this binding preventing the movement of the NAD^+^ domain associated with opening of the catalytic cleft [21]. Because the space where GTP binds becomes accessible only when the catalytic mouth is closed, GTP binds in the closed conformation only [30]. Genetic analyses of patients with the HI/HA syndrome showed that such patients harbor mutations in the regulatory domain of hGDH1 that attenuate GTP inhibition. Specifically, a number of such mutations are found in the ascending α-helix of the antenna and in the small α-helix of the descending random coil as described above. Mutagenesis studies have also confirmed that these mutations impair the interaction of GTP with hGDH1 [31,33]. There is evidence that GTP inhibits GDH1 by stabilizing abortive complexes, thus retarding product release [21,34]. On the other hand, ADP is shown to bind at Arg463 [20], located at the beginning of the pivot helix (just behind the antenna). Indeed, substitution of Ala for Arg463 is shown to abolish ADP activation. Binding of ADP at this site, which occurs both in the closed and open conformation, is thought to facilitate the NAD^+^ domain rotation, thereby reducing the energy required for opening the catalytic cleft [31]. This is consistent with the view that ADP activates the enzyme by in part enhancing product release [21,34,53].

L-leucine also activates mammalian GDH by facilitating product release [53,54], but this effect may not be mediated allosterically. Instead, indirect evidence suggests that L-leucine binds at the active site, with mutations in hGDH1 that diminish basal activity (<1.0% of capacity) (Ser409Arg, Arg443Ser, Lys450Glu and His454Tyr) shown to abrogate L-leucine activation [55]. These mutations are thought to promote the closed conformation, which may prevent L-leucine from entering the active site [55]. Observations showing that low concentrations of ADP (10–25 μM), sufficient to increase the basal activity of the Arg443Ser mutant to the baseline level of the wild-type hGDH2 (about 4% of maximal), permit L-leucine to activate these mutants are consistent with this hypothesis [28]. Moreover, these findings have provided a mechanistic explanation for the responsiveness of the wild type hGDH2 to L-leucine activation.

As noted above, steroid hormones interact with hGDH2 with a much greater affinity than with hGDH1 [51,56,57]. Thus, inhibitory assays, performed under baseline conditions, revealed that diethylstilbestrol (DES) (IC_50_ = 0.08 ± 0.01 μM) and 17 beta-estradiol (IC_50_ = 1.67 ± 0.06 μM) inhibit hGDH2 with ~ 18-fold higher affinity than hGDH1 (IC_50_ = 1.53 ± 0.24 μM for DES and IC_50_ = 26.94 ± 1.07 μM; *p* < 0.001 for 17 beta-estradiol) [56]. Also, estriol, progesterone, and pregnenolone (the precursor of all steroid hormones) inhibit hGDH2 with a 10-fold greater affinity than hGDH1 [56]. Similarly, the androgens DHEA and DHT interact with hGDH2 with a 10-fold and 25-fold greater affinity than with hGDH1 in the absence of ADP (baseline conditions) [57]. However, ADP at 0.1–1.0 mM attenuates this inhibitory effect [56,57]. In contrast, corticosterone inhibits both human isoenzymes with a similar high potency [57]. Of the various evolutionary amino acid changes acquired by hGDH2, the Arg443Ser substitution was found to be largely responsible for the sensitivity of hGDH2 to estrogens [56]. Functional analyses of wild type of hGDH1 and hGDH2 suggest that interaction of steroid hormones with these enzymes is inversely related to the state of enzyme activation induced by ADP [56]. Similarly, study of hGDH1/2 mutants, displaying various basal activities, revealed that the affinity of steroid hormones for these enzymes correlates inversely with their basal catalytic activity (R = 0.99; *p* = 0.0001) [56].

Additional studies utilizing recombinant human isoenzymes revealed that the green tea polyphenol EGCG inhibits hGDH2 with a greater affinity than hGDH1 with this differential effect being more pronounced in the absence of ADP [46]. Comparison of EGCG inhibitory curves obtained in different buffer compositions shows that both human isoenzymes are substantially more sensitive to EGCG in phosphate pH 7.50 buffer than in TRA pH 8.0 buffer [46]. Also, spermidine, an endogenous polyamine thought to reduce oxidative stress and to prolong survival, interacts with hGDH2 more potently than with hGDH1 [46]. However, in contrast to EGCG, spermidine’s inhibitory effect is stronger in TRA than in phosphate buffer. Also, increased ADP concentrations do not substantially affect spermidine inhibition. Interestingly, the hGDH2 inhibitory curve of spermidine in phosphate buffer is highly sigmoidal with Hill plot analyses suggestive of a high level of co-operative behavior (Hill co-efficient: 6.0, which is the theoretical maximum for a hexamer). As the spermidine levels in mitochondria are estimated to vary from 1.0–8.0 mM, this polyamine could exert a steady inhibitory effect on human GDHs with this effect being stronger for hGDH2 than for hGDH1. Because hGDH2 is resistant to negative modulation by GTP inhibition, regulation of this enzyme may in part be achieved by the opposing effects of ADP and endogenous inhibitors such as spermidine and steroid hormones.

Sirtuin 4 (SIRT4), a human mitochondrial ADP-ribosyltransferase, has been recently shown to inhibit GDH1 activity by using NAD^+^ to ADP-ribosylate the enzyme in the mitochondria [58]. Accordingly, knocking down SIRT4 in mice results in GDH1 activation [59]. Because a low calorie feeding downregulates SIRT4, it has been suggested that attenuation of SIRT4 activity induced by low caloric intake activates GDH in pancreatic β-cells. In addition to regulatory mutations in hGDH1, amino acid substitutions in the short-chain 3-hydroxyacyl-CoA dehydrogenase (SCHAD) can also lead to protein-sensitive hypoglycemia [60]. Recent enzymatic analysis showed that the wild-type SCHAD interacts with the wild-type hGDH1 attenuating its activity [61]. Loss of this negative modulation in patients with SCHAD deficiency leads to hyperinsulinism through hGDH1 activation [61]. These observations provide additional evidence that glutamate flux through GDH is regulated by diverse cellular mechanisms.

## 5. GDH Subcellular Localization

The subcellular localization of mammalian GDH has been studied extensively using a variety of experimental approaches. Early work using subcellular fractionation methods [62,63,64] revealed that liver GDH localizes to the mitochondrial matrix. Subsequent ultrastructural IHC studies in rat brain [65,66] have largely confirmed these findings. However, in some mammalian tissues membrane-bound GDH forms have been described [67,68,69]. GDH activity has also been recovered from nuclear fractions of rat liver [70], rat brain [71] and chicken liver [72]. More recent IHC studies in human brain revealed hGDH1 and hGDH2-specific immunostaining of the nuclear membrane of glial and neuronal cells, respectively [73]. Regarding lower organisms, the potential nuclear localization of GDH has been suggested for *S. cerevisiae* [74] but not for *D. melanogaster* [75].

The recent cloning of *GLUD1* and *GLUD2* genes, encoding hGDH1 and hGDH2, has permitted the use of molecular biological methods in order to study their subcellular destination in cultured cells. Whereas hGDH1 and hGDH2 are, in their mature form, highly homologous (98% amino acid sequence similarity), their predicted mitochondrial targeting sequences (MTS) (53 amino acid long N-terminal peptides) show a lower degree of amino acid sequence similarity (83%). This could theoretically affect their subcellular localization. To address this issue, Mastorodemos et al. [76] and Rosso et al. [77] used the enhanced green fluorescent protein (EGFP) method [78] to study the intracellular destination of hGDH1 and hGDH2. For this, *GLUD1*-*EGFP* or *GLUD2*-*EGFP* were constructed and used to transfect five mammalian cell lines (COS-7, HeLa, HEK 293, CHO and neuroblastoma SH-SY5Y). Viewed under confocal microscopy, both hGDH1-EGFP and hGDH2-EGFP were found inside the mitochondria of the transfected cells [76]. In addition, a rather minor localization to the endoplasmic reticulum was observed [76]. On the other hand, Rosso et al. [77] obtained evidence that the MTS of hGDH2 provides an enhanced mitochondrial targeting capacity. To study the mitochondrial transport of the two human proteins, Mastorodemos et al. [76] used the *ΔGLUD1-EGFP* and *ΔGLUD2-EGFP* constructs that lacked the N-terminal 53-amino acid cleavable MTS (N53), to transfect the above cell lines. They observed that removal of the MTS blocks the entrance of hGDH1 and hGDH2 into mitochondria [76,79,80].

## 6. Transport of Human GDHs in *S. Cerevisiae* Mitochondria

The budding yeast *S. cerevisiae* has been extensively used as a model organism for studying fundamental aspects of eukaryotic cell biology. A very recent study showed that ~47% of human homologue proteins can replace their yeast homologues [81]. Regarding mitochondria biology, *S. cerevisiae* has been used for exploring mitochondrial biogenesis in the human given the similarity of the mitochondrial import pathways between yeast and humans [82]. In addition, mitochondria isolated from *S. cerevisiae* have been extensively used to study the transport of nuclear-encoded mammalian proteins into these organelles. Kalef-Ezra et al. [80] have accordingly used the yeast mitochondrial import system to study aspects of mitochondrial import of hGDH1 and hGDH2. Results showed that the in vitro synthesized ^35^S-labelled hGDH1 and hGDH2 can be efficiently imported and proteolytically processed into isolated pure mitochondria from yeast *S. cerevisiae* [80]. This import depends on time and on the mitochondrial inner membrane potential. Kinetic import analysis revealed that hGDH2 is imported or/and processed slightly faster than hGDH1 [80]. These results give credence to suggestions of Rosso et al. [77] that hGDH2 is more efficiently targeted to mitochondria than hGDH1. Moreover, the hGDH1/2 MTS alone is able to import non-mitochondrial proteins (such as enhanced green fluorescent protein and dihydrofolate reductase (DHFR)) into human and yeast mitochondria [80]. Hence, these results suggest that human GDH MTS has evolved to facilitate the rapid transport of the nascent enzyme across mitochondrial membranes. The MTS of hGDH1 and hGDH2 differ from those of other proteins by their unusually large size (53 amino acid long) and the complexity of their structure. The reason for this complexity is not readily apparent; however, it may provide the efficiency needed for the fast transport of a large number of hGDH1/2 molecules in short time, thus ensuring that the enzyme reaches very high levels in mitochondrial matrix (about 10 mg/mL matrix). Modeling of hGDH1 and hGDH2 MTS predicts that they tend to form at least two distinct amphipathic α-helical structures (α1: residues 1–10 & α2: 16–32 residues) separated by short loops [79,80]. Selective deletion of the α1 helix of hGDH2 abolishes the mitochondrial import of this protein [79,80]. The α1 helix, but not the α2 helix, has an autonomous mitochondrial targeting capacity, as it can efficiently drive the non-mitochondrial proteins, such as DHFR and EGFP, in mitochondria [80]. Moreover, the fused peptide α1α2 (α1 fused with α2 without the intermediate loops), but not the α1 peptide alone, is able to target the mature hGDH2 protein into mitochondria [80]. Therefore, the mitochondrial targeting of hGDH2 relies on the synergistic effect of the two α-helical structures, with the α1 helix having the leading role [80].

Whereas the MTS of nuclear encoded mitochondrial proteins lack a common motif, these are often rich in positively charged amino acids; they are characterized by amphipathicity and a tendency to form α-helix structures [83,84]. Site-directed mutagenesis studies in hGDH2 showed that the net positive charge of the N-terminal part of the 53 amino acid long MTS of hGDH2, rather than the amphipathicity or propensity for α-helix formation, is the main determinant for their mitochondrial import [80]. Moreover, the proteolytic removal of the hGDH1 and hGDH2 MTS in the mitochondrial matrix is not necessary for their efficient mitochondrial import [80]. The nuclear-encoded mitochondrial matrix precursors are imported into mitochondria through the TOM and TIM23 translocases of the outer and inner mitochondrial membrane, respectively. Interestingly, recent studies, involving overexpression of hGDH1 in the neurons of mice, revealed a differential upregulation of TOMM20 [85]. This protein is part of the TOM channel involved in the recognition and import of precursor proteins from the cytosol into mitochondria as noted above [86,87,88]. These results suggest that the need to import large numbers of GDH molecules into the mitochondria of the host drives the over-expression of a mitochondrial translocase.

## 7. Expression of GDH in Mammalian Organs

The GDH activity contained by different mammalian tissues is known to vary widely [62,88,89]. Organs with high enzyme levels include the liver, brain, kidney, pancreas, adrenals and placenta [1,62,66,89,90,91]. The highest GDH specific activity is found in the liver [62,88,89], where the enzyme constitutes about 1% of the total protein. Other mammalian tissues contain lower GDH activities [62,88,89]. It has been estimated that the GDH content of human brain and kidneys is about 20%–25% of that of human liver [90]. Recently, Spanaki et al. [91] used antibodies that recognize both human GDH isoenzymes to study human tissues by IHC. They found that GDH is very densely expressed by all hepatocytes of human liver. In human pancreas, GDH is expressed by all cells of pancreatic parenchyma, including the acinar cells and the endocrine cells of the Langerhans islets. In human renal cortex, GDH localizes predominantly to the epithelial cells of the proximal convoluted tubules [46,91]. In human testis GDH is found in the Sertoli cells and in the Leydig cells [90,91]. On the other hand, the germ cells (spermatogonia, spermatocytes and spermatozoa) are negative for GDH [90,91].

## 8. Expression of hGDH1 and hGDH2 in Non-Neural Human Tissues

The development of antibodies specific for either hGDH1 or hGDH2 made it possible to study the specific expression of each isoenzyme in human tissues [90,92]. In human liver, a very dense expression of hGDH1 is detected in all hepatocytes within cytoplasmic structures resembling mitochondria. In contrast, human hepatocytes are essentially devoid of hGDH2-specific immunoreactivity. Other cells of the hepatic parenchyma are negative for hGDH1 or hGDH2. These include the endothelial cells that line the sinusoids and the bile tubules (canaliculi, ductules and interlobular bile ducts) [57]. The very dense hGDH1 staining of hepatocytes is in accord with previous observations derived from enzymatic assays showing that the highest levels of GDH activity occur in the liver. Furthermore, the absence of hGDH2 staining in human liver is in accord with mRNA data showing that human liver does not express the *GLUD2* gene [23]. In human kidney, the epithelial cells lining the proximal convoluted tubules of the renal cortex densely express both hGDH1 and hGDH2 with a similar pattern. Also, the epithelial cells of the distal convoluted tubules and of the collecting ducts stain positively for hGDH1 and hGDH2, but this staining is less intense than that observed in the proximal convoluted tubules. In contrast, no hGDH1 or hGDH2 immune-reactivity was detected in other cells of the human kidney [46,92]. In human adrenal cortex all three layers that produce steroid hormones express densely both hGDH1 and hGDH2. These include the zona glomerulosa, the zona fasciculata and the zona reticularis that secrete mineralocorticoids, glucocorticoids and androgens, respectively. Also, the sympathetic neurons located inside the adrenal medulla or in the sympathetic ganglia stain positively for both hGDH1 and hGDH2. In contrast, the chromaffin cells of the adrenal medulla are negative for the two human isoenzymes. In human ovaries, both hGDH1 and hGDH2 are expressed by all hormone-producing ovarian cells. In contrast, cells lacking endocrine function express the hGDH1 isoenzyme only. Specifically, hGDH1 and hGDH2 are both expressed in the granulosa cells that surround the oocyte in primordial follicles or in primary follicles. In tertiary follicles both human GDH isoenzymes are expressed by the androstenedione-producing luteinized cells of the theca interna. On the other hand, the follicular granulose cells that are known to synthesize 17 β-estradiol show lower levels of hGDH1 and hGDH2 expression. However, ovarian cells that lack endocrine function, located in the theca externa and in the ovarian stroma (undifferentiated cells), express the hGDH1 isoenzyme only [57]. In human placenta, intense punctate immunoreactivity for both hGDH1 and hGDH2 is found in the syncytiotrophoblast cells that produce progesterone and estriol, two female hormones essential for the maintenance of pregnancy and fetal development. In contrast, the endothelial cells that line the fetal capillaries, constituting the core of the placenta villi, express hGDH1 only. In human testis, a dense expression of hGDH2 was observed in the Sertoli cells that support the developing sperm cells. In contrast, the Sertoli cells are not labeled with the hGDH1-specific antibody. On the other hand, the Leydig cells, which are known to synthesize androgens, express both hGDH1 and hGDH2. The immature germ cells (spermatogonia, spermatocytes and spermatozoa) are negative for both human GDH isoenzymes. In human prostate, all cells of the prostatic gland show intense punctate hGDH1 labeling. In contrast, prostatic cells are completely devoid of hGDH2 immunoreactivity. Hence, whereas in human kidneys and adrenals hGDH1 and hGDH2 co-exist in the same cells, in human liver, ovaries, placenta, testis and prostate the cellular expression of hGDH1 and hGDH2 is distinct.

## 9. Expression of GDH in Nerve Tissue of Experimental Animals

Previous studies have revealed that GDH1 activity is heterogeneously distributed among synaptic and non-synaptic mitochondria derived from different CNS regions of experimental animals. Thus, Leong and Clark [93] showed that the GDH specific activity of nonsynaptic mitochondria from medulla oblongata and pons of the rat is about two-fold higher than those from the striatum or from the cortex. In contrast, the GDH specific activity of synaptic mitochondria does not show a similar regional variation. Also, ultrastructural studies by Aoki et al. [65] revealed a marked variation in the mitochondria content of GDH in rat brain as described below. Rothe et al. [94] showed that GDH specific activity is low in the nervous system of the rat at the time of birth, but during the postnatal period this increases by 5.2 fold in the hippocampal formation and by 2.3 fold in the cerebellar cortex. These changes are thought to reflect synaptogenesis and maturation of glutamatergic structures. Zaganas et al. [95] measured GDH specific activities in extracts from cultured neurons and astrocytes prepared from mouse cerebral cortex and cerebellum and found that cultured astrocytes contain higher GDH activity as compared to cultured neurons. Interestingly, the GDH specific activity of the glutamatergic cerebellar granule cells is about 60% higher (*p* < 0.01) than that of GABAergic cerebral cortical neurons. Granule cells are thought to need the GDH anaplerotic function for synthesizing their transmitter glutamate [95].

Aoki et al. [65,96] used IHC with light microscopy and immunogold electron microscopy to study the cellular and regional distribution of GDH in rat brain. They found that GDH is mainly expressed by astrocytes, the regional distribution pattern of which corresponds to that of glutamatergic pathways. Astrocytic GDH staining is significantly different from that of the GFAP, a marker for astrocytes. Thus, in rat hippocampus, GDH is detected in the superficial layers of the AC (associational commissural pathway) and within the stratum lacunosum-moleculare in close association with the walls of blood vessels. In contrast, GFAP staining shows a more widespread distribution throughout all hippocampal layers. In the striatum, intense GDH astrocytic staining is observed in the nucleus accumbens and ventral pallidum. Similarly, the nucleus basalis of Meynert and the basolateral amygdaloid complex show dense GDH astrocytic staining. Whereas the labeling pattern of GDH and GFAP in the hippocampus differ markedly, the two proteins co-localize in the medulla oblongata, with the nucleus of the solitary tract being heavily labeled. Additional areas with significant GDH immunoreactivity include the ventral thalamic nuclei, the sensory relay nuclei, the inferior olives, the pontine nuclei, the efferent transverse fibers of the pons, the subthalamic nucleus and the substantia nigra. In contrast to astrocytes, neuronal cells show rather low intensity GDH staining only when Triton X-100 is omitted from the labeling procedure.

Wenthold et al. [97] also studied by IHC rat cerebellum and observed intense labeling of Bergmann glial cell bodies and fibers. Also, astrocytes in the granule cell layer were heavily labeled. Whereas a light immunereactivity was detected in Purkinje and granule cell bodies, the stellate, basket and Golgi cells did not stain. Rothe et al. [66] using both pre-embedding immunocytochemical staining and post-embedding immunogold labeling and quantitative ultra-structural methodology demonstrated that GDH1 is densely expressed in the Bergmann glia. Also, protoplasmic astrocytes in the granular layer and fibrous astrocytes in the white matter of the rat cerebellar cortex were similarly labeled. In contrast, cerebellar neurons show very low GDH1 expression with neuronal mitochondria exhibiting about 15% of the GDH1 specific staining detected in astrocytic mitochondria. Finally, a low, but specific, GDH1 staining was also observed in the white matter within the mitochondria of cells of oligodendrocytic origin. Schmitt and Kugler [98] studied the cellular and regional expression of GDH1 in the rat cerebellum, hippocampus and spinal cord at the mRNA level (using non-radioactive in situ hybridization) and at the protein level (using IHC with an affinity purified antibody raised against bovine GDH1 protein). A strong signal for GDH1 mRNA was detected in glial cells (Bergmann glia, astrocytes and their processes) in the neuropil and around blood vessels, forming the outer limiting membrane. Less intense, but still significant, in situ hybridization signal was detected in neurons, oligodendrocytes, ependymal cells and the epithelial cells of the choroid plexus. However, IHC revealed that the GDH1 protein is expressed only in astrocytes and Bergmann glia. On the other hand, neurons are devoid of significant GDH1 expression, except for the neurons of spinal ganglia [98].

## 10. Expression of hGDH1 and hGDH2 in Human Cerebral Cortex

**Figure 3:**
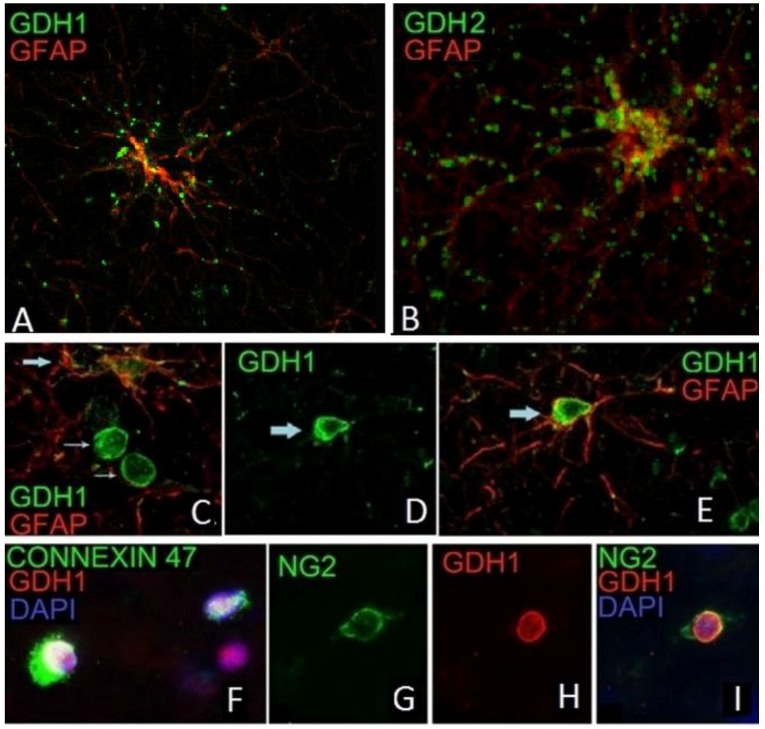
hGDH1 and hGDH2 expression in glial cells. Punctate immunoreactivity for hGDH1 (**A**) and hGDH2 (**B**) is present in the cytoplasm and along the proximal and distal processes of GFAP positive astrocytes (IF images of high magnification). Also, hGDH1-specific staining is detected in the nucleus of a GFAP positive astrocyte (**C,D,E**), of a connexin 47-positive oligodendrocyte (**F**) and of an NG2-positive oligodendrocyte-precursor (**G,H,I**) (IF images of unfixed human frontal lobe cortex. Blue staining represents DAPI labeled nuclei).

Using double immunofluorescence with the anti-hGDH1 or the anti-hGDH2 specific antibodies and specific cellular/subcellular markers, Spanaki et al. [16,71,90] studied the expression of these proteins in human cortical tissues. Results revealed that both hGDH1 and hGDH2 are expressed in GFAP positive astrocytes within coarse cytoplasmic structures consistent with mitochondria (Figure 3). Both the perikarya of cortical astrocytes and their proximal and distal processes are labeled. The hGDH1 and hGDH2 positive astrocytes are distributed in both gray and white matter of human cortical tissue. Also, hGDH1 specific immunoreactivity was identified in the nuclear membrane of the vast majority of the oligodendrocytes and their NG2-positive precursors but only in a minority of human cortical astrocytes (Figure 3). Double IF studies revealed that hGDH1 co-localizes with lamin A/C to the nuclear membrane of oligodendrocytes.

**Figure 4:**
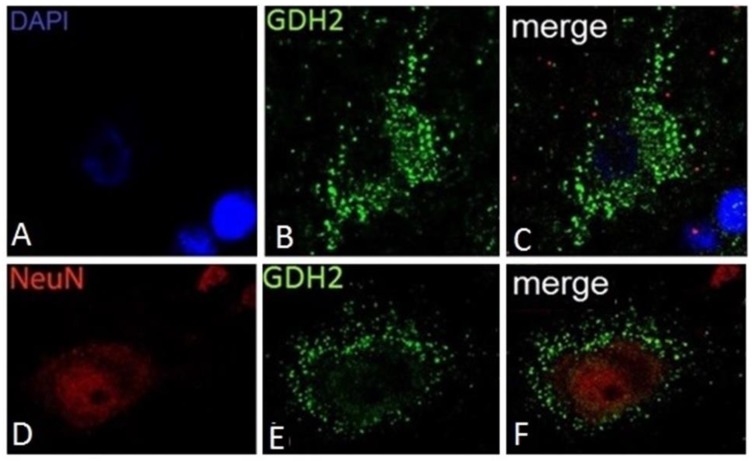
Localization of hGDH2-positive “puncta” in the periphery and in the perinuclear cytoplasmic area of large human cortical neurons. Specific hGDH2 punctate staining is observed in the cytoplasm of a large neuron from the occipital lobe within coarse structures resembling mitochondria (**A**–**C**). A constellation of intense hGDH2-specific “puncta” is also detected in the periphery of a sizable frontal lobe neuron with a large nucleus and prominent nucleolus (**D**–**F**). (IF images of unfixed human frontal and occipital lobe cortex. Blue staining represents DAPI labeled cell nuclei).

**Figure 5:**
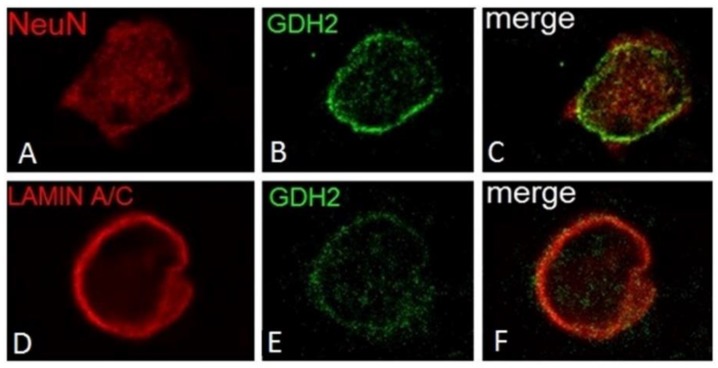
Association of hGDH2 with the nuclear membrane in small cortical neurons. A delicate circular GDH2 specific-staining is detected around the NeuN-positive area (**A**–**C**). Double IF using anti-hGDH2 and anti lamin A/C specific antiserums revealed that hGDH2 co-localizes with Lamin A/C on the nuclear membrane of this cell (**D**–**F**). (IF images of unfixed human frontal lobe cortex).

Regarding human cortical neurons, these do not show significant hGDH1-specific labeling, in accordance with the results of previous studies on rat brain described above. In contrast, the anti-hGDH2 antibody labels large cortical neurons with pyramidal cell morphology (Figure 4). In some of these large neurons, the hGDH2-specific antibody stains coarse cytoplasmic structures (resembling mitochondria) dispersed throughout the cell body (perikaryon) and in the axon (Figure 4). In other pyramidal neurons, however, large hGDH2-positive “puncta” were observed in the cell periphery (Figure 4). Double IF studies further revealed that these dense hGDH2-specific “puncta” are on the cell surface in close proximity to GFAP-positive astrocytic endfeet that delineate the neuronal cell membrane. In addition, the anti-hGDH2 antibody labels small round neurons with dense nuclei (Figure 5). In these neurons, hGDH2-specific staining was detected in the nuclear membrane, where it co-localizes with lamin A/C (Figure 5) [16].

## 11. Functional Roles of the GDH Pathway in Various Tissues

Whereas the role of GDH in cell biology is still incompletely understood, there is increasing evidence that different cells utilize the glutamate flux through the GDH pathway in order to carry out some of their unique functions [16]. Consistent with this concept are results of the IHC studies described above showing that a heterogeneous and multifaceted distribution pattern of hGDH1 and hGDH2 exists in human tissues. These observations support the possibility that the functional role of the GDH pathway varies from tissue to tissue and even between cellular systems of the same organ.

In human liver, the exceptionally dense expression of hGDH1 in periportal and perivenous hepatocytes, permits these cells to accomplish multiple metabolic tasks, including ammonia metabolism and acid/base homeostasis [99,100,101,102]. Hepatocytes are exposed to variable concentrations of glutamate and/or ammonia, substrates expected to drive the GDH reaction towards the breakdown or synthesis of glutamate, respectively. Krebs originally suggested that the high levels of GDH in hepatocytes (10% of matrix proteins) keep the enzyme’s reactants in equilibrium [100]. The bi-directionality of the GDH pathway in the liver has been confirmed by labeling experiments [103,104].

Whereas GDH1 in the liver can reach equilibrium, in tissues with normally low ammonia concentrations, the high K_m_ for ammonia (20–30 mM) may prevent the enzyme from proceeding towards the reductive amination direction, particularly under conditions of acidosis as described above. In pancreatic β-cell cells glutamate flux through the GDH1 pathway is linked to insulin secretion through ATP synthesis [33]. Consistent with this view are observations of Stanley et al. [33] on patients with regulatory mutations in hGDH1 that render the enzyme overactive. In these subjects, an increased oxidative deamination of glutamate by the overactive hGDH1 results in increased release of insulin from pancreatic β-cells, resulting in hypoglycemia. Whereas activation of pancreatic GDH1 by L-leucine leads to insulin secretion, GDH1 also provides an amplification pathway for the full development of glucose-stimulated insulin release. Thus, islets isolated from mice lacking β-cell GDH1 exhibited 50% lower insulin secretion rates than islets from control mice. This amplification pathway may assume importance for glucose homeostasis when high calorie feeding prevails or in states of insulin resistance. Under conditions of high calorie intake, islets of wild-type mice exhibit enhanced insulin responses, whereas islets of GDH1 knockout mice fail to increase their insulin secretion [105,106]. Moreover, knock-out mice maintained on high calorie diet exhibit reduced fat storage in their adipocytes, as compared to the wild-type mice [106]. Whether this is due to the function of GDH1 in lipogenesis, as discussed above, or to a different mechanism, is presently unclear.

As described above, hGDH1 and hGDH2 are densely expressed in the steroid synthesizing cells of the various human steroidogenic organs. At present, the functional role of these proteins in steroid-producing cells needs to be further understood. However, there is a close association between the cellular distribution of hGDH1/2 and that of the cholesterol side chain system involved in steroid biosynthesis, the early stages of which require reducing equivalents (NADPH) [57]. It has been accordingly suggested the glutamate flux through hGDH1 and hGDH2 may generate NADPH (Figure 1) needed for steroid biosynthesis [57]. As the housekeeping hGDH1 and the novel hGDH2 are regulated by distinct mechanisms, their co-expression in human steroid-producing cells may permit glutamate flux under changing energy demands as also noted below for astrocytres. This needs to be further studied.

In human testis, the dense expression of hGDH2 in the Sertoli cells is thought to contribute to the supportive role of these cells in nourishing the spermatids [57,90]. As Sertoli cells provide spermatids with lactate and other nutrients, the parallelism has been drawn between Sertoli cells and astrocytes, also known to provide neurons with lactate. Sonnewald et al. [107] have showed that ^13^C-labeled glutamate is oxidatively metabolized (via GDH) in astrocytes with a significant proportion of the label found in lactate (Figure 1). Whereas the expression of hGDH2 in Sertoli cells is thought to be advantageous by permitting glutamate metabolism under conditions of adequate energy supply, the absence of hGDH1 expression in human Sertoli cells is poorly understood. On the other hand, expression of hGDH1 and hGDH2 in Leydig cells is thought to contribute to biosynthesis of androgens produced by these cells [57].

In human kidney, GDH is believed to be involved in the regulation of acid-base homeostasis, particularly under acidotic conditions. Thus, while under normal acid-base states the enzyme remains relatively idle, during acute or chronic acidosis renal GDH is activated and its expression is up-regulated. In kidney, GDH operates towards the deamination direction, contributing to renal ammonia production by at least 25% [37]. Although enhanced GDH function does not significantly increase total nitrogen excretion, it is crucial for the efficient handling of metabolically produced acids. Specifically, GDH provides an “emergency” pathway for ammonia production under acidotic conditions, thus permitting the kidney to efficiently regulate acid-base homeostasis. The role of GDH in the production of renal ammonia is underlined by observations on patients with the HI/HA syndrome due to mutations that attenuate GTP inhibition [33]. The overactive hGDH1 enzyme in these patients has been shown to enhance the renal production of ammonia resulting in hyperammonemia [108]. In contrast to hypoglycaemia that may develop after a meal enriched in L-leucine, hyperammonemia is continuous, not related to dietary protein [108]. As noted above, both human isoenzymes (hGDH1 and hGDH2) are expressed in the epithelial cells lining the proximal convoluted tubules of the renal cortex, but the reason(s) for this is not well understood. It is, however, likely that the specific properties of the hGDH2 enzyme (lower optimal pH, resistance to GTP inhibition), permit renal hGDH2 to function under conditions that are inhibitory for hGDH1 [109].

In brain, the normally high glutamate levels along with the normally low ammonia concentrations are thought to drive the hGDH1 reaction towards the oxidative deamination direction. This is supported by results of labeling studies using 13N as a tracer [110]. Even under conditions of hyperammonemia, such labeling studies [111] have revealed little incorporation of 13N-labeled ammonia into brain glutamate. As described above, hGDH1 is densely expressed in glial cells (astrocytes, oligodendrocytes and their precursors) but not in neurons [90]. On the other hand, hGDH2 is expressed in both glial and neuronal cells. At the subcellular level, hGDH1 and hGDH2 localize to the mitochondria of astrocytes and of large cortical neurons, respectively. In addition, hGDH1 localizes to the nuclear membrane of glial cells and hGDH2 to the nuclear membrane of small cortical neurons (Figure 2 and Figure 4) [73]. These observations suggest that, in addition to the well-known role of hGDH1/2 in mitochondrial metabolism, these enzymes may also be involved in nuclear processes.

Early studies by Berl et al. [112] revealed that glutamate metabolism in brain is compartmented, with this compartmentalization thought to facilitate the dual role of glutamate in metabolism and in neuronal transmission processes [113]. Specifically, localization of GDH in astrocytes is thought to permit these cells to handle high quantities of glutamate released from nerve terminals during excitatory transmission [114]. As noted above, Aoki et al. [65] indeed obtained evidence that in rat brain GDH is densely expressed in the mitochondria of astrocytes, the regional distribution pattern of which corresponds to that of the glutamatergic pathways. Synaptic astrocytes are known to remove more than 80% of the glutamate released from the presynaptic glutamatergic nerve endings during excitatory transmission [114]. There is evidence that glutamate taken up by astrocytes is in large part oxidized by the Krebs cycle [115,116]. Once inside the mitochondria, glutamate can enter the Krebs cycle as α-ketoglutarate via oxidation by GDH or transamination by ATT2. As compared to ATT2, the function of which requires another Krebs cycle substrate, GDH is anaplerotic. It appears that glutamate flux through GDH is driven by high extracellular glutamate levels (similar to those that occur in synapses during intense excitatory transmission) [116]. The functional role of GDH and ATT in glutamate metabolism in astrocytes, synaptic mitochondria and brain slices was recently reviewed by McKenna et al. [117]. This topic was also recently reviewed by Cooper and Jeitner [118]. Studies on transgenic mice lacking brain *Glud1* (Cns*Glud1*-/-) revealed altered metabolic handling (decreased oxidation) of glutamate [119]. Also, inhibition of GDH expression by siRNA in cultural astrocytes results in a dysfunctional TCA cycle [120]. Transgenic mice without brain GDH (Cns*Glud1*-/-) show an energy-deprivation state in their hypothalamus that alters the energy homeostasis of the whole-body [121].

Whereas both hGDH1 and hGDH2 are expressed in human cortical astrocytes, the role of the two human isoenzymes in the biology of these cells is not well understood. However, as the two isoenzymes possess distinct energy sensors and exhibit different optimal pHs, their co-expression may enhance the ability of astrocytes to metabolize neurotransmitter glutamate under changing energy demands and cellular acidification [17]. Thus, in contrast to hGDH1, the energy sensor of which allows glutamate to fuel the Krebs cycle when cellular energy levels decline, hGDH2 is not subject to GTP control and, therefore, can operate efficiently under conditions of an adequate cellular energy charge. Whether hGDH2 evolution, driven by positive selection, has contributed to the marked expansion of astrocytic arborization in the human (as compared to rat brain) it needs to be further studied.

Expression of hGDH2 in human cortical neurons, including glutamatergic nerve terminals is thought to enhance the formation of pre-synaptic glutamate thus strengthening cortical excitatory transmission [73]. Consistent with this possibility are observations on transgenic mice with targeted over-expression of GDH1 in their cortical neurons, showing increased pre-synaptic glutamate release [122]. Also, during neuronal activation in human subjects, glutamate levels increase in the cortex via enhanced GDH flux [123]. Whereas the mechanism by which hGDH2 expression in nerve terminals could promote glutamate formation is presently unclear, it is likely that oxidative deamination of glutamate (derived from glutamine deamidation in mitochondrial matrix) generates α-ketoglutarate, which is then transferred (via the α-ketoglutarate/malate transporter) to the cytosol where it is transaminated to glutamate [124].

In addition to its putative function in brain glutamatergic transmission, hGDH2 may play a role during nerve tissue development and maturation, probably by promoting lipid biosynthesis. Thus Li et al. [125] recently studied transgenic mice that carry in their genome the human *GLUD2* gene and observed transcriptome changes that parallel the differences found between humans and macaques. The authors also described that the metabolic effects of the transgenic *GLUD2* expression observed were around the Krebs cycle. The maximal *GLUD2* effects were found during early post-natal development, suggesting the possibility that *GLUD2* expression may support lipid biosynthesis through hGDH2-linked oxidative generation of citrate (Figure 1) as originally suggested for glioma cells [126].

The implications of hGDH1 expression in the nuclear membrane of a subpopulation of astrocytes and of the vast majority of oligodendrocytes and oligodendrocyte precursors (NG2-positive cells) remain unclear. However, it has been recently realized that α-ketoglutarate, the substrate of GDH, serves as co-factor for α-ketoglutarate-dependent dioxygenases, nuclear enzymes involved in DNA and histone deacetylation [127]. Dividing cells maintain high levels of α-ketoglutarate needed for their replication processes [128]. In light of these considerations, it is of interest that hGDH1 is expressed in the nucleus of glial cells capable of proliferation. These include protoplasmic astrocytes and oligondendrocyte precursors (NG2 positive cells) that are known to differentiate to mature oligodendrocytes. On the other hand, expression of hGDH2 in small cortical neurons is perplexing.

## 12. Involvement of the GDH Pathway in Human Disorders

Altered function or deregulation of GDH is shown to be involved in several human diseases. The best known paradigm is the HI/HA syndrome caused by regulatory mutations in the *GLUD1* gene that render the enzyme resistant to GTP inhibition [33]. Over-activity of hGDH1 in pancreatic β-cells leads to enhanced insulin secretion through increased ATP synthesis [30,33]. As a result, patients harboring such mutations develop hypoglycemia following a protein rich meal. They often exhibit CNS symptoms such as epileptic seizures, mental retardation, and generalized dystonia [33,129]. The pathogenesis of epileptic fits has not been fully understood, although there is evidence that they may relate to alterations in energy metabolism and glutamate excitotoxicity rather than to abnormal neuronal discharge [130]. There are even suggestions that GDH deregulation is involved in other forms of epilepsy. Thus, decreased GDH activity has been found in the temporal cortex and hippocampus of patients with mesial temporal lobe epilepsy [131]. On the other hand, GDH activity is reported to be increased in the active epileptogenic areas of cerebral cortex of patients with generalized and focal epilepsies [132]. There is also evidence that drugs with convulsive or anticonvulsive properties target GDH function [133,134,135]. Regarding the pathogenesis of dystonia, observed in patients with regulatory mutations in the *GLUD1* gene, this is thought to result from chronic GABA depletion [136,137]. Specifically, over-activity of mutant hGDH1 may deplete the glutamate pool involved in GABA synthesis, thus attenuating GABA-mediated inhibition.

Recently, deregulation of hGDH2 function was found to influence Parkinson’s disease (PD) onset [135]. Thus, a rare DNA polymorphism (T1492G) in the regulatory domain of hGDH2 that results in replacement of Ser445 by Ala is shown to affect the age at PD onset. Specifically, male hemizygotes for the T1492G developed Parkinson disease 6–13 years earlier than individuals without this genotype [138]. This effect was not found in female PD patients who were heterozygous for T1492G change. Functional analyses of the Ala445-hGDH2, obtained in recombinant form, revealed that the mutant enzyme exhibits enhanced catalytic activity thus suggesting that deregulation of glutamate metabolism accelerates ongoing PD neurodegeneration [138]. As female hormones interact potently with the overactive Ala445-hGDH2, it is possible that inhibition of the variant enzyme by estrogens may protect heterozygous females [138]. Also, observations on transgenic (Tg) animals genetically engineered to overexpress hGDH1 in their neurons, have provided additional evidence that hGDH1 deregulation is associated with accelerated neurodegeneration. Thus, Tg animals over-expressing neuronal hGDH1 exhibit age-dependent degeneration of the CA1 hippocampal region resembling Alzheimer’s disease pathology [122]. Hence, hGDH1 and hGDH2 have emerged as important players in the pathogenesis and treatment of human diseases related to glutamate dysfunction. These include some of the common degenerative neurological diseases for which effective treatments remain to be discovered.

## 13. Involvement of hGDH1 and hGDH2 in the Biology of Glioma and Other Neoplasias

Almost a century ago, Warburg showed that tumor cells use alternate pathways to resist metabolic stress. This metabolic reprogramming is presently considered as the defining characteristic of cancer cells. When glucose metabolism is insufficient, glutaminolysis supports the viability of cancer cells by contributing ATP and anabolic carbons for the biosynthesis of amino acids, nucleotides, and lipids [139,140,141,142,143]. During glutaminolysis, glutamine is catabolyzed by glutaminase to glutamate, which is then converted to α-ketoglutarate by either the GDHs (GDH1 and GDH2) or by the transaminases (GOT2 and GPT2). There is increasing evidence that GDH is the predominant pathway for the synthesis of α-ketoglutarate from glutamate. Indeed, several studies have shown that GDH expression is up-regulated in cancer cell lines and in tissues from patients with different neoplasias, including gliomas, leukemias, breast, lung and colorectal cancers [3,144,145,146,147]. Moreover, GDH expression levels correlate with poor outcomes, tumor size and metastatic disease [3,146,147]. This may be due to the enzyme’s role both in bioenergetics [147] and in redox homeostasis [3]. Jin and co-workers [3] have found that GDH1 is predominantly responsible for the conversion of glutamate to α-ketoglutarate in lung cancer H1299 cells and in breast cancer MDAMB231 cells lines. The enzyme confers a significant advantage to tumor development, with GDH1 knockdown in lung and cancer breast cells found to reduce cell proliferation and tumor growth [3]. GDH1 is shown to regulate the intracellular levels of α-ketoglutarate and its subsequent metabolite fumarate, which in turn activates glutathione peroxidase 1, thus regulating redox homeostasis and tumor growth. Inhibition of GDH1 by shRNA or the purpurin analogue R162 affects redox homeostasis impairing cell proliferation and tumor growth. Zhang et al. [147] have made similar observations in human glioma cells. Yang et al. [148] showed that GDH activity is upregulated in SF188 glioblastoma cells grown under conditions of impaired glycolysis and that inhibition of GDH by EGCG enhances the sensitivity of these cells to drugs that target glucose metabolism.

While research into the glioma biology has uncovered numerous molecular alterations, some of which are in core biological pathways of oncogenesis, the cause of the initial neoplastic transformation remains unclear. On the other hand, somatic mutations in IDH, an enzyme that inter-converts isocitrate to α-ketoglutarate, were recently identified as occurring early in gliomatogenesis, with 70%–90% of low grade glioma and secondary glioblastoma multiforme found to harbor such mutations [149,150,151,152]. Other tumors, such as acute myeloid leukemia, cholangiocarcinoma, chondrosarcoma, and melanoma are found to harbor somatic IDH mutations at lower frequency rates (about 20%) [149,151,152]. Mutations in IDH markedly impair the ability of the enzyme to convert isocitrate to α-ketoglutarate. Instead, α-ketoglutarate is aberrantly reduced by the mutant IDH to D-2-hydroxyglutarate, which accumulates at high concentrations in glioma cells. In turn, D-2-hydroxyglutarate acts as an oncometabolite by competitively inhibiting dioxygenases, nuclear enzymes that use α-ketoglutarate to demethylate DNA and histone. Because mutant IDH cannot convert isocitrate to α-ketoglutarate, the neoplastic cells need to generate α-ketoglutarate by upregulating alternative metabolic pathways in order to maintain normal levels of critical metabolites, including NADPH. Indeed gene expression profiling revealed that *GLUD1* and *GLUD2* that generate α-ketoglutarate via glutamate oxidation, are selectively and consistently up-regulated in glioma cells harboring IDH mutations. Moreover, studies in glioma cells with the R132H IDH1 mutation revealed that selective inhibition of *GLUD2* expression markedly slows cell growth [126]. Conversely, expression of *GLUD2* (but not *GLUD1*) promotes tumor expansion, suggesting that R132H IDH1 glioma cells proliferate by utilizing enhanced glutamate flux through the *GLUD2* pathway [126]. Furthermore, labeling studies in glioma progenitor cells revealed that glutamate flux through hGDH2 provides α-ketoglutarate for oxidative generation of citrate through the Krebs cycle, thus promoting lipid biosynthesis [126]. Hence, by regulating bioenergetics and redox homeostasis human GDH1/2 have emerged as key players in the pathogenesis of human neoplasias and as novel therapeutic targets for halting tumor development and expansion.

## 14. Conclusions

Glutamate dehydrogenase is a hexameric enzyme that catalyzes the reversible conversion of glutamate to α-ketoglutarate and ammonia while reducing NAD(P)^+^ to NAD(P)H. GDH is found in all living organisms, with lower life forms often expressing distinct GDH isoenzymes with strict specificity for NAD^+^ or NADP^+^. In contrast, mammalian GDH1 (hGDH1 in the human) shows dual co-enzyme specificity, being able to utilize both NAD^+^ and NADP^+^ for catabolic and synthetic reactions, respectively. Oxidative deamination of glutamate by GDH1 in mitochondria generates α-ketoglutarate that is then metabolized by the Krebs cycle leading to ATP synthesis. This permits glutamate to replace glucose as energy source. GDH1 attains very high levels in the liver (10% of mitochondrial matrix proteins) where it is involved in multiple metabolic processes, including ammonia and acid-base homeostasis. In addition to hGDH1, humans and other primates have acquired via duplication an hGDH2 isoenzyme, which underwent rapid evolutionary selection concomitant with human brain development. The novel isoenzyme is not expressed in human liver. Instead, hGDH2 is expressed, along with hGDH1, in human cerebral cortex, kidney, testis and steroid producing organs. However, the two isoenzymes show a distinct cellular distribution pattern. Thus, in human cerebral cortex, hGDH1 localizes mainly to mitochondria of astrocytes and the nuclear membrane of oligodendrocytes and their precursors. In contrast, hGDH2 localizes to the mitochondria of large cortical neurons and astrocytes, and in the nuclear membrane of small cortical neurons. Whereas the functional implications of the nuclear localization of hGDH1/2 are presently unknown, α-ketoglutarate is recently shown to serve as co-enzyme for α-ketoglutarate-dependent nuclear dioxygenases. In astrocytes, GDH is involved in the metabolism of transmitter glutamate and the production of lactate. In human testis, hGDH2, but not hGDH1, is densely expressed in the Sertoli cells, which are known to support the spermatids by providing them lactate and other nutrients. In adrenals, ovaries and placenta, hGDH1 and hGDH2 are densely expressed in steroid producing cells. In these steroidogenic organs, the two human isoenzymes are specifically expressed in the cells that synthesize steroid hormones, where they may provide NADPH for the synthesis of these hormones. Recently, evidence for additional functions for hGDH1/2 has emerged. Thus, in glioma progenitor cells, hGDH2 is shown to provide α-ketoglutarate for lipid biosynthesis through oxidative generation of citrate. In this regard, recent data showing that hGDH2 is highly operational during rapid brain growth are thought to suggest that hGDH2 supports early nervous system development by promoting lipid biosynthesis. Lastly, in neoplastic cells hGDH1 is shown to regulate redox homeostasis via generation of fumarate. Hence, different cells may utilize the GDH flux for accomplishing some of their unique functions.
